# Light at a tunnel’s end: The lightwand as a rapid tracheal location aid when encountering false passage during tracheostomy

**DOI:** 10.4103/0972-5229.74173

**Published:** 2010

**Authors:** Umesh Goneppanavar, Shwethapriya Rao, Nanda Shetty, Prabhu Manjunath, Daniel Thomas Anjilivelil, Sadasivan S. Iyer

**Affiliations:** **From:** Department of Anaesthesiology, Kasturba Hospital, Kasturba Medical College, Manipal, India

**Keywords:** Decannulation, distorted anatomy, false passage, lightwand, open tracheostomy

## Abstract

False passage and loss of airway during tracheostomy are not uncommon, especially in patients with short and thick necks. Distorted neck anatomy following either repeated insertion attempts or due to underlying malignancy may make it very difficult to locate the trachea even while attempting open/surgical tracheostomy, despite good exposure of the neck in such situations. The lightwand is not an ideal device for tracheal intubation in such patients. However, it can be useful in these patients while performing open tracheostomy. Passing the lightwand through the orotracheal tube can aid in rapid identification of the trachea in such situations and may help reduce the occurrence of complications subsequent to repeated false passage. We report a series of four such cases where use of lightwand aided in rapidly locating the trachea during tracheostomy complicated by distorted anatomy.

## Introduction

A lighted stylet/lightwand uses the principle of transillumination of soft tissues of the anterior neck to guide the tip of a tracheal tube into the trachea. It takes advantage of the anterior or more superficial location of the trachea in relation to the esophagus.[1] Lighted stylets have become accepted tools in airway management in various clinical scenarios.[2] Recently, it has been shown that lighted stylets are not only helpful in rapid and reliable tracheal intubation, but also help in percutaneous tracheostomy. Use of a lightwand can avoid the risk of puncturing the endotracheal tube and/or cuff, thus allowing adequate ventilation and oxygenation during percutaneous tracheostomy.[3] The incidence of difficulty in securing open (surgical) tracheostomy has been reportedly higher in individuals with short and thick necks.[4] Although the lightwand is not the ideal device for intubation in such patients, it can be useful in these to locate the trachea while performing open tracheostomy.

## Case Report

**Figure 1:**
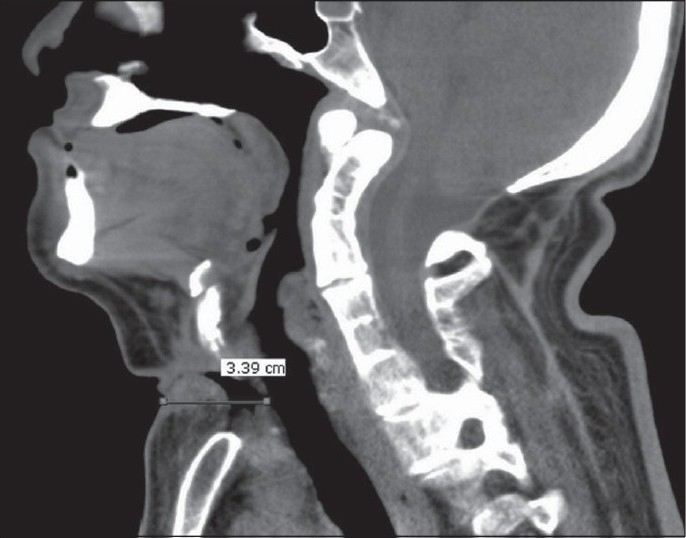
MRI neck lateral view showing thick and short neck with restricted neck extension due to fused cervical vertebrae and the depth of the trachea from skin level to be 3.4 cm

**Figure 2:**
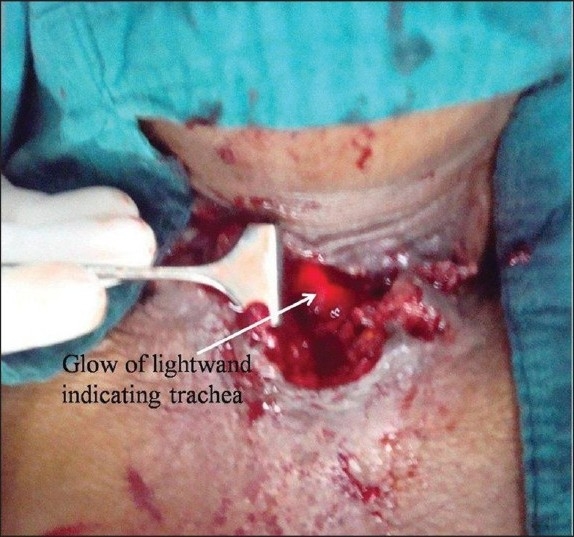
Neck picture of case 4, showing distorted location of trachea and identification of the trachea by the glow of the lightwand

Tracheostomy was complicated by the creation of a false passage or distorted anatomy due to underlying malignancy in four adult patients. In the first two patients, a false passage was created during insertion of the tracheostomy tube. The false passage had resulted in displacing the trachea much more posteriorly than its usual location [Figure 1] and hence it was difficult for the surgeons to locate the trachea while attempting a repeat tracheostomy. The lightwand was useful in accurately identifying the trachea, following which tracheostomy could be revised easily. In the third patient, although the trachea was identified with the aid of a lightwand, we could not negotiate the tracheostomy tube, which seemed to be due to narrowing of the tracheal stoma, as a smaller size tracheostomy tube was inserted successfully with the aid of a lightwand in the critical care unit itself. In this case, the lightwand was used not only to locate the trachea but also as an aid for railroading the tracheostomy tube. In the fourth patient, distorted anatomy due to underlying malignancy had resulted in difficulty in locating the trachea, which was solved by the use of a lightwand [Figure 2]. Following these successes, we have successfully reinserted the dislodged tracheostomy tubes in our critical care unit with the aid of a lightwand on a couple of occasions.

## Discussion

A tracheostomy is an opening or stoma created in the trachea to aid in breathing/ventilation and toileting of pulmonary secretions. Tracheostomy can be performed by two different approaches, namely, open surgical tracheostomy (ST) and percutaneous dilational tracheostomy (PDT). Irrespective of the technique used, multiple problems can occur during and immediately after a tracheostomy. Passing the tracheostomy tube into the stoma during tracheostomy or during unscheduled replacement of dislodged tracheostomy tubes can be a challenging procedure for the physician and a frightening experience for the patient.[5] Forceful attempts at replacement can be dangerous and can lead to disruption of the soft tissues adjacent to the tracheostomy tract, resulting in a false passage. Accidental extubation of the tracheostomy tube and creation of a false passage on re-insertion are not uncommon events.[6] Accidental decannulation during change of tie has been reported in the literature, which requires appropriate precautionary measures.[6] Increased tracheal mucosal swelling and increased tissue friability have been identified as the major risk factors for creation of a false passage during tracheostomy.[7] Reported incidence of false passage is around 11 and 160 per 10,000 procedures with ST and PDT, respectively.[8] There are many suggested measures to minimize the risk of creating a false passage while restoring a lost airway during tracheostomy. They are use of a fiberoptic bronchoscope, nasogastric tube, digital feel of the tracheostomy stoma and insertion of a jet ventilation/airway exchange (JVAE) catheter through the endotracheal tube prior to surgical entry into the trachea through a bronchoscope port attachment.[9,10] Utility of lightwands in assisting percutaneous tracheostomy is well established.[3] Although using the fingertip to palpate the trachea and endotracheal tube is a useful technique to locate the tip of the tracheal tube during the procedure, it is unreliable, particularly in patients with a short or thick neck.[3]

Although there is literature evidence to show that a lightwand is useful in percutaneous tracheostomy for locating the trachea, there is only one report of its utility in open tracheostomy.[4] This case series is the first report of the utility of a lightwand in rapidly locating the trachea in revision tracheostomy that is complicated by distorted anatomy following false passage or malignancy.

Therefore, based on our experience, we suggest that the lightwand can be a useful device for securing open tracheostomy and should be used whenever available as an aid to locate the trachea correctly. Furthermore, we believe that the routine use of a lightwand to locate the trachea when faced with the initial incidence of a false passage in critical care units can effectively reduce the occurrence of complications such as pneumomediastinum, and injury to vessels or nerves in the neck.
